# MRI of non-specific low back pain and/or lumbar radiculopathy: do we need T1 when using a sagittal T2-weighted Dixon sequence?

**DOI:** 10.1007/s00330-019-06626-6

**Published:** 2020-02-04

**Authors:** Fabio Zanchi, Raphaël Richard, Mahmoud Hussami, Arnaud Monier, Jean-François Knebel, Patrick Omoumi

**Affiliations:** 1 Department of Diagnostic and Interventional Radiology, Lausanne University Hospital and University of Lausanne, Rue du Bugnon 46, 1011 Lausanne, Switzerland; 2 Department of Radiology, Riviera-Chablais Hospital, Avenue de la Prairie 10, 1800 Vevey, Switzerland; 3 Laboratory for Investigative Neurophysiology (The LINE), Department of Radiology, University Hospital Center and University of Lausanne, 1011 Lausanne, Switzerland; 4 EEG Brain Mapping Core, Centre for Biomedical Imaging (CIBM), 1011 Lausanne, Switzerland

**Keywords:** Magnetic resonance imaging, Low back pain, Radiculopathy, Spine, Cost savings

## Abstract

**Objective:**

To show that for the MRI workup of non-specific low back pain and/or lumbar radiculopathy, the acquisition of T1-weighted sequences in the sagittal plane could be waived when using an FSE T2-weighted Dixon sequence.

**Materials and methods:**

Three musculoskeletal radiologists retrospectively reviewed fifty lumbar spine MRI examinations performed for non-specific low back pain and/or lumbar radiculopathy. Two protocols were separately analyzed in the sagittal plane: a standard protocol (T1-weighted, in-phase, and water-only images of an FSE T2-weighted Dixon sequence) and a simplified protocol (fat-only, in-phase, and water-only images of an FSE T2-weighted Dixon sequence). Eight items usually assessed on T1-weighted sequences were analyzed for each of the vertebrae (*n* = 250), vertebral endplates (*n* = 500), vertebral corners (*n* = 1000), foramina (*n* = 500), lamina (*n* = 500), and facet joints (*n* = 500). Interchangeability of these protocols was tested using the individual equivalence index. A decrease in interobserver agreement of ≥ 5% when one reader used the simplified protocol compared with when both readers used the standard protocol was considered clinically significant. Interreader and intrareader agreement were assessed using kappa statistics. Rates of findings with each protocol were compared using odd ratios.

**Results:**

The standard and simplified protocols were interchangeable (range of upper bound of the 95%CI of individual equivalence index = 0.25 to 1.38%). Intraprotocol and interprotocol interreader kappa values were similar (0.253–0.671 vs. 0.236–0.723, respectively). Rates of findings were not statistically significantly different (*p* ≥ 0.074), or were higher with the simplified protocol (*p* ≤ 0.036).

**Conclusion:**

In our target population, a single sagittal T2-weighted Dixon sequence may replace the recommended combination of T1-, T2-, and fat-suppressed T2-weighted sequences.

**Key Points:**

*• In patients with non-specific low back pain or lumbar radiculopathy, spine MRI in the sagittal plane could be limited to a single FSE T2-weighted Dixon sequence, hereby reducing the acquisition time.*

*• A simplified protocol of spine MRI in the sagittal plane combining FSE T2-weighted Dixon sequence provides the same information as a standard protocol including T1-, T2-, and fat-suppressed T2-weighted sequences for the workup of degenerative lumbar spine lesions.*

*• For some findings shown on the simplified protocol, such as focal bone marrow replacement lesions or signs of infection, additional sequences including pre- and post-contrast T1-weighted sequences may be required, as is currently the case when using the standard protocol.*

**Electronic supplementary material:**

The online version of this article (10.1007/s00330-019-06626-6) contains supplementary material, which is available to authorized users.

## Introduction

Non-contrast magnetic resonance imaging (MRI) is considered the best imaging method to investigate low back pain when conservative treatment fails or when red flags, indicating an underlying cause of the pain, are present [1, 2]. Red flags have been defined by ACR Appropriateness Criteria and include history of cancer, cauda equina syndrome, or signs of infection [1, 2]. In their absence, low back pain is qualified as non-specific [2]. The lifetime prevalence of low back pain is estimated to be between 38.9% worldwide, and depending on the country, a significant proportion of these patients will undergo lumbar spine MRI, making this examination one of the most commonly performed MRI examinations worldwide [2–4].

While acquisition protocols of lumbar spine MRI may vary widely between institutions, it is generally recommended to include spin-echo T1-weighted, T2-weighted, and fat-suppressed fluid-sensitive sequences in the sagittal plane [5–7]. The protocol may further include acquisitions in the transverse plane, targeted on the intervertebral levels that show structural abnormalities in the sagittal plane.

Recently, there has been a growing interest in the use of the Dixon fat suppression technique combined with fast spin-echo (FSE) acquisitions for various musculoskeletal applications, including spine MRI [8–15]. The Dixon technique presents some advantages compared with other fat suppression methods. Firstly, the Dixon technique provides more homogeneous fat suppression than the chemical shift selective (CHESS) technique, which is particularly useful in large field-of-view acquisitions such as for the spine or in the presence of metal. Secondly, it provides higher signal-to-noise ratio than short-tau inversion recovery (STIR) techniques [8, 9, 16–18]. Finally, the Dixon technique has the advantage of producing, in a single acquisition, four sets of images with different contrasts, which has been advantageously used to reduce examination time by waiving the need to acquire some standard sequences, such as for the imaging of sacroiliitis, spinal metastases, or the knee [10–13]. Specifically, a Dixon T2-weighted sequence generates in-phase (similar to standard T2-weighted images), out-of-phase, fat-only, and water-only images (corresponding to fat-suppressed T2-weighted images) [19–21]. Therefore, a single Dixon T2-weighted sequences provides the combination of non-fat-suppressed and fat-suppressed fluid-sensitive sequences that is recommended in the context of low back pain/lumbar radiculopathy [10]. Furthermore, as previously shown for the detection of focal bone marrow lesions in patients with suspected metastases, fat-only images could theoretically replace T1-weighted sequences for the assessment of the fatty components of the spine [5, 9, 13, 22]*.*

We hypothesized that in the workup of non-specific low back pain/lumbar radiculopathy, the acquisition of T1-weighted sequences in the sagittal plane could be waived when using FSE T2-weighted Dixon sequences. Therefore, our primary aim was to show, in the context of non-specific low back pain and lumbar radiculopathy, the interchangeability of a simplified protocol containing a single sagittal FSE T2-weighted Dixon sequence and a standard protocol containing sagittal FSE T1-weighted and T2-weighted Dixon sequences. Our secondary aims were (1) to show that interreader agreement when both readers used the standard protocol was similar to the interreader agreement when one reader used the simplified protocol and (2) to compare the rates of findings using the standard and the simplified protocols.

## Materials and methods

### Study population

**Fig. 1:**
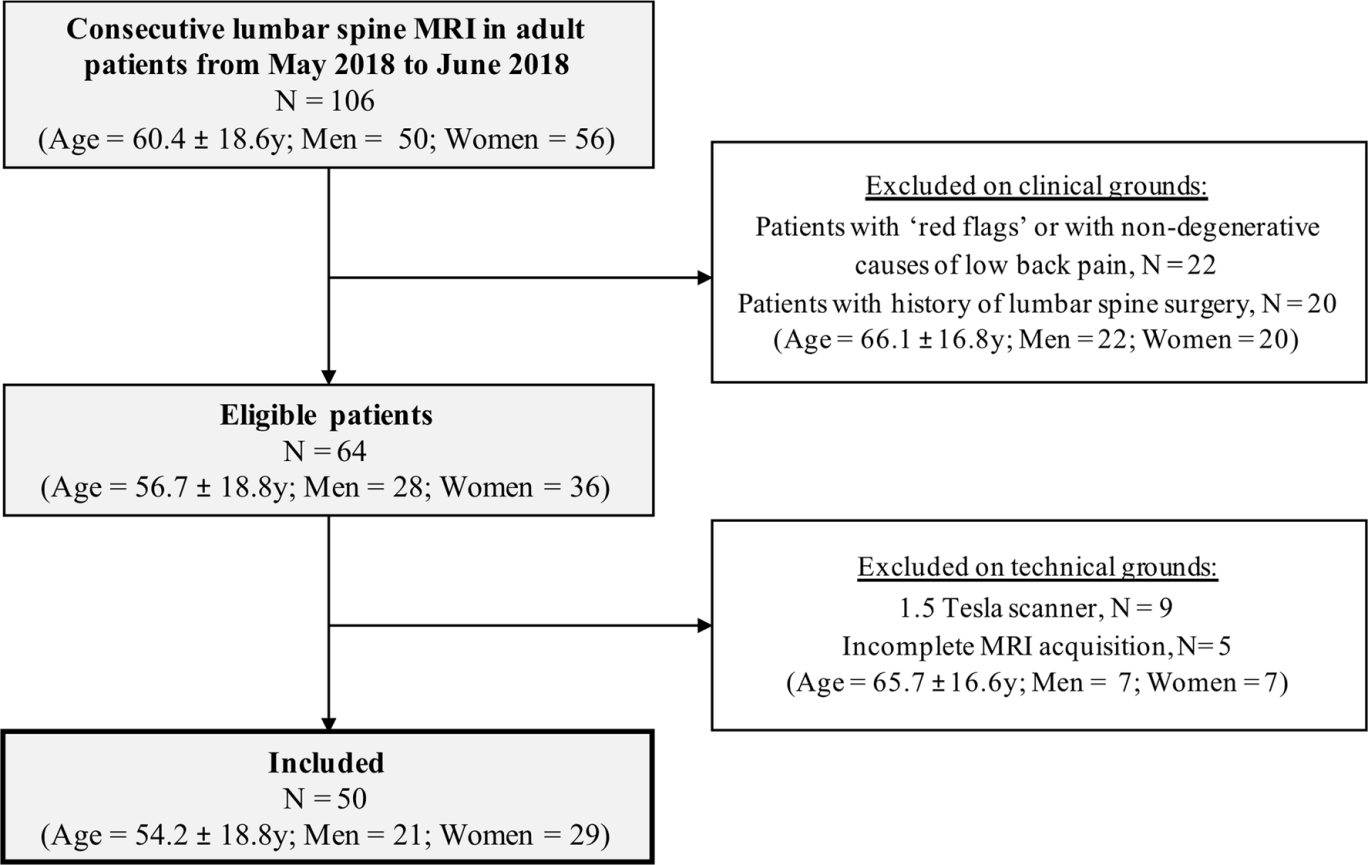
Flowchart shows selection criteria and patient characteristics

Our single-center study was approved by our institutional ethics committee (Swiss Ethics Committees on research involving humans, Project ID 2017-01555), which did not require informed consent because of the retrospective design. We retrospectively included consecutive adult patients undergoing lumbar spine MRI at our institution for low back pain, radicular lumbar pain, or both, from May 2018 to June 2018 (Fig. 1). We excluded all examinations performed on patients for whom red flags as defined by the ACR Appropriateness Criteria were present, or for whom the cause of the pain was clinically related to non-degenerative disorders (*n* = 22; including 10 history of cancer, 7 acute trauma, 5 osteoporotic fractures) [1]. We also excluded patients with a history of recent lumbar spine surgery (*n* = 20), as well as examinations performed on a 1.5-T scanner (*n* = 9) or with incomplete acquisitions (*n* = 5). It is to note that the sagittal T2-weighted Dixon sequences were part of our acquisition protocol prior to this study and were used to provide homogeneous fat suppression.

### MRI examinations

All MRI examinations were performed on commercially available 3-T scanners (Somatom Skyra, Skyra Fit, Prisma; Siemens) without hardware modifications, using the scanner’s radiofrequency body coils. The imaging parameters of the sequences of interest are detailed in Table 1. All examinations included an FSE T1-weighted and an FSE T2-weighted two-point Dixon sequences on the lumbar spine. Four sets of images were routinely reconstructed from the FSE T2-weighted Dixon sequences: in-phase, out-of-phase, water-only, and fat-only. Additional sequences, such as contrast material–enhanced fat-suppressed T1-weighted sequences and sequences in the axial plane, which could have been added on a case-by-case basis, were not considered for this study.

**Table 1:** MRI acquisition parameters

Parameter	FSE T1-weighted sequence	FSE T2-weighted Dixon sequence
Plane	Sagittal	Sagittal
No. of sections	19–30	19–30
Section thickness (mm)	3–3.5	3–3.5
Gap (mm)	0.3–0.7	0.3–0.7
Field of view (mm)	260 × 260–300 × 300	260 × 260–280 × 280
Acquisition matrix	384 × 230–448 × 358	320 × 192–320 × 280
Phase-encoding direction	Head to feet	Head to feet
Repetition time/echo time (ms)	418–946/11–14	3230–6850/80–94
Turbo factor	4	18
No. of averages	1–2	1–2
IPAT factor	2–3	2–3
Phase oversampling	0.6–0.9	0.6–1
Flip angle (degrees)	120–160	121–150
Bandwidth (Hz/pixel)	210–211	340
Acquisition time: mean ± standard deviation; range (min:s)	2:50 ± 0:31; 1:22–4:14	3:59 ± 0:41; 2:07–6:05

*iPAT*, integrated parallel acquisition technique

In total, the acquisition time of the standard protocol was 6:49 ± 0:65, range 4:29–9:58 (vs. 3:59 ± 0:41, range 2:07–6:05, for the simplified protocol)

### Image review

Three musculoskeletal radiologists, from two institutions, with 1, 2, and 4 years of experience (MH, AM, RR) independently analyzed two protocols: the standard protocol (consisting of a sagittal T1-weighted sequence, as well as the in-phase and water-only images of the sagittal T2-weighted Dixon sequence) and the simplified protocol (consisting of the in-phase, water-only, and fat-only images of the sagittal T2-weighted Dixon sequence). The protocols were read in a random order, separately, during different sessions at least 3 weeks apart, on the institution’s PACS workstations (Vue PACS, Carestream Health Inc.). The findings for each examination and each protocol were recorded on standardized diagrams (submitted as supplementary material) and transferred to spreadsheets. Readers were blinded to clinical data and to each other’s assessments. There was no training session prior to the study.

**Fig. 2:**
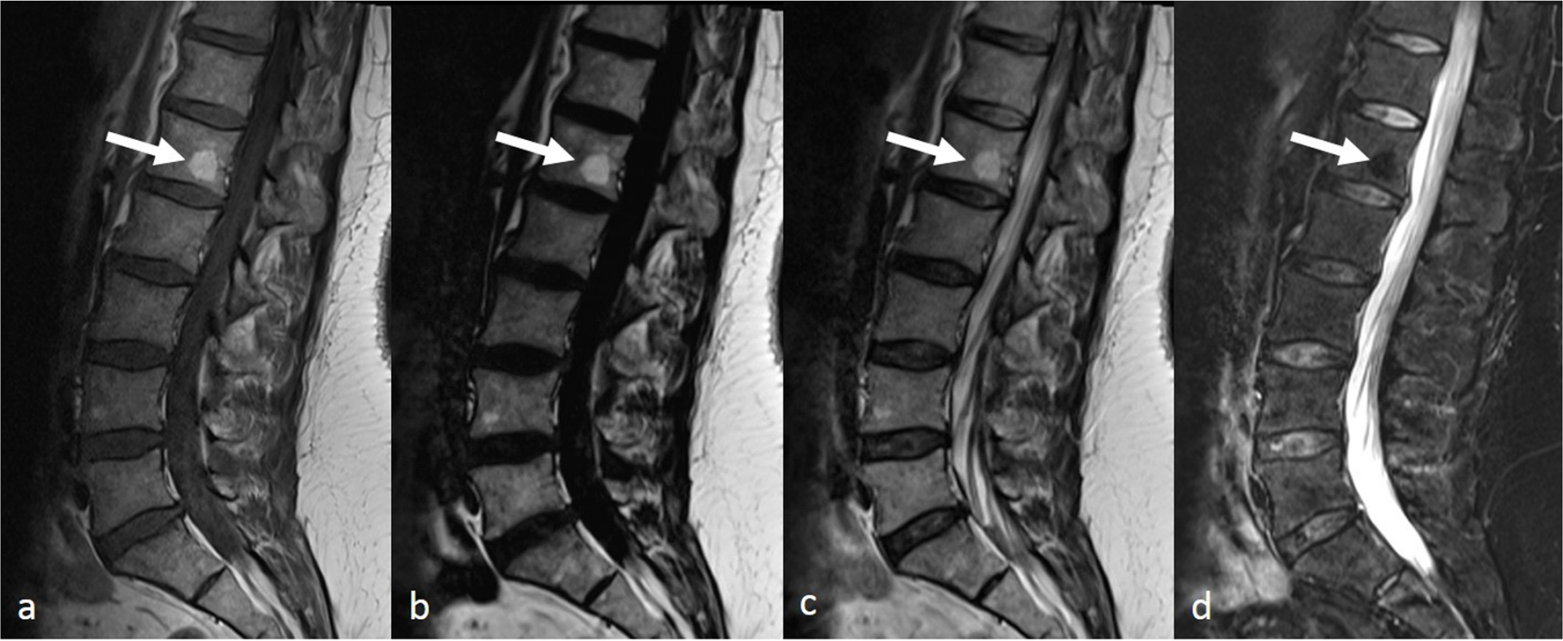
Lumbar spine MRI in a 41-year-old female including T1-weighted (**a**), fat-only image (**b**), in-phase image (**c**), and water-only image (**d**) from T2-weighted Dixon sequence. A focal hyperintense bone marrow area is visible on the L1 vertebral body on **a** (arrow), also clearly visible on **b**, with signal suppression on **d**, compatible with an area of focal area of red marrow depletion, due to a fatty hemangioma or a fat island

**Fig. 3:**
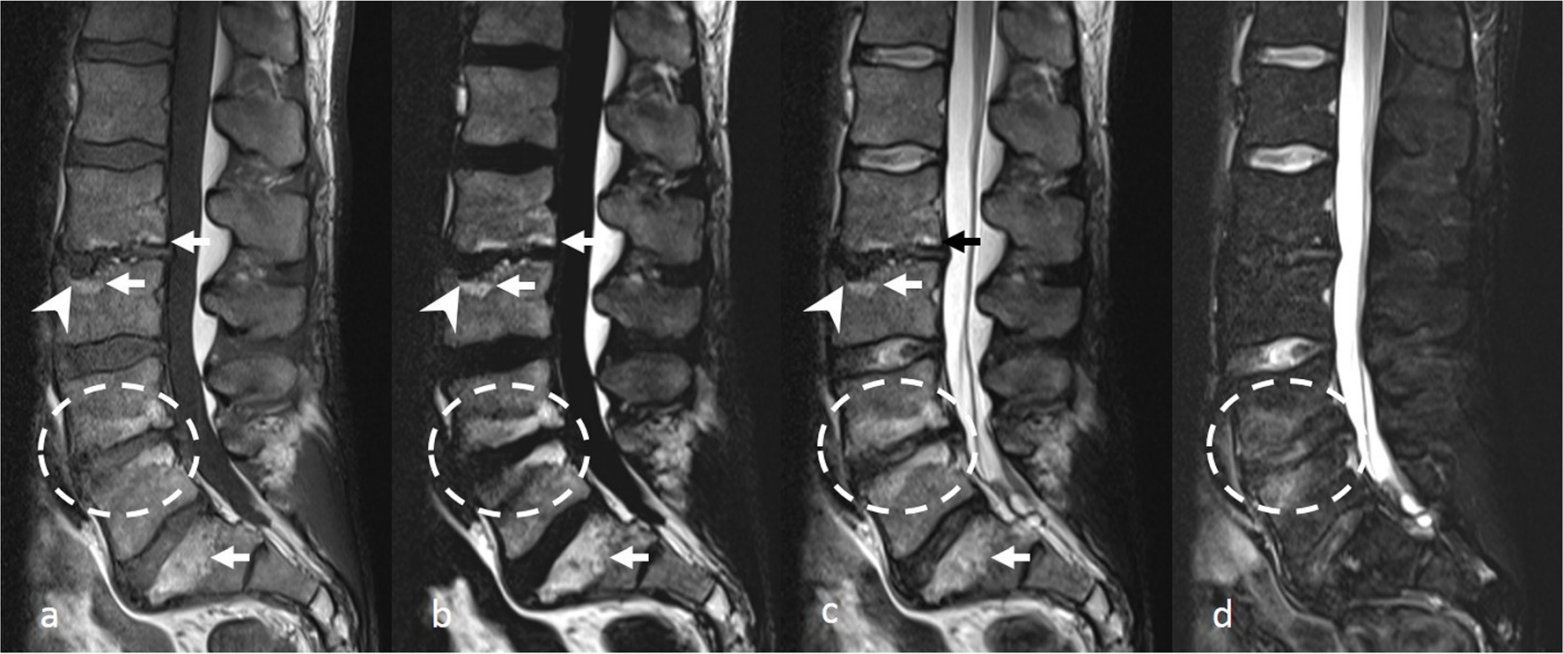
Lumbar spine MRI in a 38-year-old male including T1-weighted (**a**), fat-only image (**b**), in-phase image (c), and water-only image (**d**) from T2-weighted Dixon sequence. Fatty Modic type 2 changes are visible along the vertebral endplates adjacent to L2-L3 disc and superior endplate of S1 on **a**–**c** (arrows). Mixed Modic changes, combining predominantly inflammatory changes anteriorly (as seen on **d**), and fatty changes posteriorly are seen along the vertebral endplates adjacent to L4-L5 disc (dashed circles). Note the high contrast between fatty changes and surrounding tissues on **b**. Schmorl’s node is seen at superior endplate of L3 vertebral body (arrowhead), well depicted on **a**–**c**

**Fig. 4:**
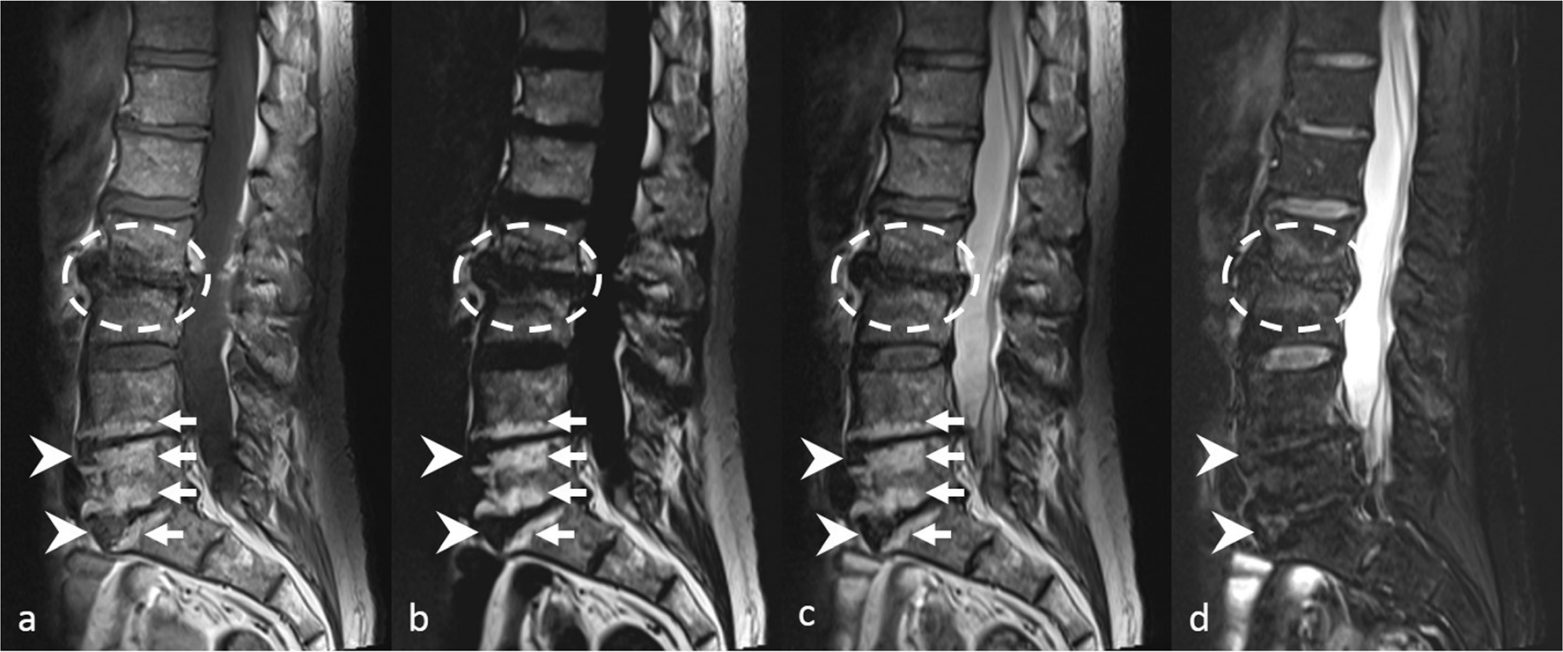
Lumbar spine MRI in a 63-year-old male including T1-weighted (**a**), fat-only image (**b**), in-phase image (**c**), and water-only image (**d**) from T2-weighted Dixon sequence. Fatty Modic type 2 changes are visible along the vertebral endplates adjacent to L4-L5 and L5-S1 discs on **a**–**c** (arrows). Degenerative changes at the anterior margins of vertebral bodies are also seen at the same levels (arrowheads). Mixed inflammatory and fatty Modic changes are seen along the vertebral endplates adjacent to L2-L3 disc (dashed circles). Note the high contrast between fatty changes and surrounding tissues on **b**

The following items, which are usually assessed on T1-weighted sequences, were evaluated according to previous descriptions from the literature: focal bone marrow abnormalities (including focal bone marrow depletion, bone marrow infiltration, bone marrow replacement, or signal void) (Fig.1) [23]; juxtadiscal Modic changes, classified as inflammatory, fatty, or fibrous according to the original publication (mixed lesions with concomitant fatty and inflammatory changes were graded separately) (Figs. 2 and 3) [24]; degenerative changes at the margin for the vertebral bodies (including fatty changes, erosions, osteophytes) (Figs. 3 and 4); Schmorl’s nodes (Fig. 2); facet arthropathy; spondylolysis and vertebral fractures which were reported as present or absent; and foraminal stenosis, graded as absent, mild, moderate, or severe, according to a previously published grading system [25]. On each examination, each lumbar vertebra (*n* = 5), vertebral endplate (*n* = 10), vertebral corner (*n* = 20), facet joint (*n* = 10), intervertebral foramen (*n* = 10), and lamina (*n* = 10) were assessed separately by each of the three readers, on each protocol.

### Statistical analysis

We tested the interchangeability of the standard and simplified protocols using the individual equivalence index [26–28]. Testing for interchangeability provides evidence that a new and a conventional imaging test achieves similar results for individual patients, which is particularly useful in situations where a reference standard cannot be obtained (such as for most items of this study) [27]. The interreader agreement rate when (1) all readers used the standard protocol (intraprotocol interreader agreement rate) was compared with the agreement rate when (2) one reader used the simplified protocol, and the other used the standard protocol (interprotocol interreader agreement rate). Interreader agreement was defined as both readers assessing each item identically, including its categorization. The interprotocol (standard vs. simplified) interreader agreement rate was subtracted from the intraprotocol (standard vs. standard) interreader agreement rate, giving the individual equivalence index, and a 95% confidence interval (95%CI) was calculated using bootstrapping methods with 1000 repetitions, i.e., by repeating the calculation of the equivalence index 1000 times based on a set of patients randomly sampled with replacement [28, 29]. To address the clustered nature of the data (several vertebrae per patient), generalized estimation equations (GEE) were used to calculate interreader agreement rates. The interchangeability of the two protocols was defined as an individual equivalence index of less than 5%, meaning that the difference in the rate of agreement between conditions (1) and (2) was considered clinically acceptable if less than 5%, which corresponds to a test of non-inferiority of the rate of interreader agreement when (2) vs. (1).

Interreader agreement was also calculated using Cohen’s kappa statistics for conditions (1) and (2), as well as for the interprotocol intrareader agreement. Unweighted kappa statistics were used for all items, except for foraminal stenosis (for which linear weighting was used). Kappa values were interpreted using the Landis and Koch grading system [30]. Finally, the rate of findings for each item in each protocol was calculated. Logistic regression models, using generalized estimation equations (GEE), were built to test for the presence of an effect related to the protocol (independent variable) on the rate of findings (dependent variable). We estimated the model parameters using generalized estimation equations (GEE) to take into account the clustered nature of the data [31]. A *p* value < 0.05 was considered statistically significant.

Statistical tests were performed using R 3.1.3 (R Core Team (2015). R: A language and environment for statistical computing. R Foundation for Statistical Computing. URL http://www.R-project.org/), MedCalc Statistical Software version 17.2 (MedCalc Software bvba; https://www.medcalc.org; 2017), and IBM SPSS Statistics for Macintosh, version 25.0 (IBM Corp.).

## Results

### Study population

A total of 50 patients were included (age = 54.2 ± 18.8 years; range 21.19–87.54 years; men = 21, women = 29) (Fig. 1), leading to 250 vertebrae; 500 endplates, facet joints, foramina, and lamina; and 1000 vertebral corners read by each of the three readers and on each of the two protocols.

### Interchangeability

Detailed results of tests of interchangeability are reported in Table 2. The intraprotocol (standard vs. standard) interreader agreement rate ranged from 1996/3000 (66.53%) (degenerative changes at margins of vertebral bodies) to 1490/1500 (99.33%) (spondylolysis), while it ranged from 4036/6000 (67.27%) (degenerative changes at margins of vertebral bodies) to 2980/3000 (99.33%) (spondylolysis) for the interprotocol (standard vs. simplified) agreement rate. The upper bound of the 95%CI for the individual equivalence index never exceeded the + 5% limit for any of the items assessed (range, 0.25 to 1.38%), indicating interchangeability between the two protocols.

**Table 2:** Intraprotocol and interprotocol interreader agreement and 95% confidence interval of the individual equivalence index (interprotocol minus intraprotocol interreader agreement)

Item	Interreader agreement rate
Focal bone marrow abnormalities	
Intraprotocol (standard vs. standard) Interprotocol (standard vs. simplified) 95% confidence interval for individual equivalence index (%)	688/750 (91.73%) 1361/1500 (90.73%) − 1.27 to 1.20
Juxtadiscal Modic changes	
Intraprotocol (standard vs. standard) Interprotocol (standard vs. simplified) 95% confidence interval for individual equivalence index (%)	1302/1500 (86.80%) 2582/3000 (86.07%) − 1.03 to 1.07
Degenerative changes at margins of vertebral bodies	
Intraprotocol (standard vs. standard) Interprotocol (standard vs. simplified) 95% confidence interval for individual equivalence index (%)	1996/3000 (66.53%) 4036/6000 (67.27%) − 1.07 to 1.01
Schmorl’s nodes	
Intraprotocol (standard vs. standard) Interprotocol (standard vs. simplified) 95% confidence interval for individual equivalence index (%)	1204/1500 (80.27%) 2406/3000 (80.20%) − 1.17 to 1.32
Endplate fractures	
Intraprotocol (standard vs. standard) Interprotocol (standard vs. simplified) 95% confidence interval for individual equivalence index (%)	1480/1500 (98.67%) 2968/3000 (98.93%) − 0.33 to 0.32
Foraminal stenosis	
Intraprotocol (standard vs. standard) Interprotocol (standard vs. simplified) 95% confidence interval for individual equivalence index (%)	1311/1500 (87.40%) 2608/3000 (86.93%) − 1.02 to 1.05
Facet arthropathy	
Intraprotocol (standard vs. standard) Interprotocol (standard vs. simplified) 95% confidence interval for individual equivalence index (%)	1104/1500 (73.60%) 2212/3000 (73.73%) − 1.32 to 1.38
Spondylolysis	
Intraprotocol (standard vs. standard) Interprotocol (standard vs. simplified) 95% confidence interval for individual equivalence index (%)	1490/1500 (99.33%) 2980/3000 (99.33%) − 0.27 to 0.25

*Note*: Total number of observations varies between items in the following manner for the standard protocol: focal bone marrow abnormalities: 50 × 5 vertebrae × 3 readers; juxtadiscal Modic changes, Schmorl’s nodes, endplate fractures, facet arthropathy, and spondylolysis: 50 × 5 levels × 2 sides × 3 readers; degenerative changes at the margins of vertebral bodies: 50 × 5 levels × 4 vertebral corners × 3 readers. For the interprotocol agreement (standard vs. simplified), the total number of observations is doubled

### Kappa statistics

Detailed results of the kappa statistics are reported in Table 3. The intraprotocol (standard vs. standard) and interprotocol (standard vs. simplified) interreader agreement were fair to substantial, ranging from 0.253 (95%CI, 0.197 to 0.309) to 0.671 (95%CI, 0.535 to 0.807) and from 0.236 (95%CI, 0.192 to 0.280) to 0.723 (95%CI, 0.632 to 0.815), respectively. The maximum difference between the intraprotocol and interprotocol interreader agreement was 0.072.

**Table 3:** Intraprotocol interreader, interprotocol interreader, and interprotocol intrareader agreement using kappa statistics

Item	Intraprotocol (standard vs. standard) interreader agreement	Interprotocol (standard vs. simplified) interreader agreement	Interprotocol intrareader agreement
Focal bone marrow abnormalities 95%CI	0.413 0.337–0.536	0.405 0.339–0.472	0.760 0.678–0.843
Juxtadiscal Modic changes 95%CI	0.393 0.332–0.454	0.389 0.348–0.431	0.781 0.735–0.826
Degenerative changes at the margins of the vertebral bodies 95%CI	0.389 0.361–0.416	0.357 0.333–0.381	0.792 0.770–0.814
Schmorl’s nodes 95%CI	0.253 0.197–0.309	0.236 0.192–0.280	0.874 0.839–0.909
Endplate fractures 95%CI	0.671 0.535–0.807	0.723 0.632–0.815	0.914 0.838–0.989
Foraminal stenosis 95%CI	0.578 0.523–0.634	0.570 0.531–0.609	0.843 0.807–0.879
Facet arthropathy 95%CI	0.362 0.316–0.407	0.359 0.323–0.396	0.716 0.676–0.755
Spondylolysis 95%CI	0.542 0.290–0.794	0.470 0.274–0.667	0.629 0.374–0.884

*Note*: All items were graded using unweighted Cohen’s kappa statistics except for foraminal stenosis (linear weight)

The interprotocol intrareader agreement was substantial to almost perfect for all items, (ranging from 0.629 [95%CI, 0.374 to 0.884] to 0.914 [95%CI, 0.838 to 0.989]).

### Rate of findings

Detailed results of the rate of findings are reported in Table 4. The rate of findings for each item ranged from 0.4 (spondylolysis) to 21% (degenerative changes at the margins of vertebral bodies). All items were reported predominantly on both protocols. For three items, a statistically significant difference was observed in favor of the simplified protocol: focal bone marrow abnormalities (16/74 (21.6%) vs. 8/74 (10.8%), *p* = 0.036); foraminal stenosis (40/237 (16.9%) vs. 15/237 (6.3%), *p* = 0.002); and facet arthropathies (118/518 (22.8%) vs. 57/518 (11%), *p* = 0.004).

**Table 4:** Rates of findings

Item	Total number of findings	Reported on both protocols	Reported on the standard protocol only	Reported on the simplified protocol only	*p* value
Focal bone marrow abnormalities, *N* = 1500	74 (4.9%)	50 (67.6%)	8 (10.8%)	16 (21.6%)	0.036*
Juxtadiscal Modic changes, *N* = 3000	214 (7.5%)	159 (74.3%)	16 (7.5%)	39 (18.2%)	0.074
Degenerative changes at the margins of the vertebral bodies, *N* = 6000	1258 (21%)	1004 (79.8%)	116 (9.2%)	138 (11%)	0.178
Schmorl’s nodes, *N* = 3000	254 (8.5%)	205 (80.7%)	18 (7.1%)	31 (12.2%)	0.103
Endplate fractures, *N* = 3000	32 (1.1%)	27 (84.4%)	4 (12.5%)	1 (3.1%)	0.199
Foraminal stenosis, *N* = 3000	237 (7.9%)	182 (76.8%)	15 (6.3%)	40 (16.9%)	0.002*
Facet arthropathy, *N* = 3000	518 (17.3%)	343 (66.2%)	57 (11%)	118 (22.8%)	0.004*
Spondylolysis, *N* = 3000	13 (0.4%)	6 (46.1%)	5 (38.5%)	2 (15.4%)	0.075

*Notes*: Lesions were considered as either present or absent

*N* represents the total number of observations for each item: focal bone marrow abnormalities: 50 × 5 vertebrae × 3 readers × 2 protocols; juxtadiscal Modic changes, Schmorl’s nodes, endplate fractures, facet arthropathy, and spondylolysis: 50 × 5 levels × 2 sides × 3 readers × 2 protocols; degenerative changes at the margins of vertebral bodies: 50 × 5 levels × 4 vertebral corners × 3 readers × 2 protocols

*p* values were calculated using logistic regression models, with generalized estimation equations (GEE) to take into account the clustered nature of the data

*Indicates a statistically significant result (< 0.05)

## Discussion

In this paper, we first showed the interchangeability of a simplified protocol (composed of images generated by a single FSE T2-weighted Dixon sequence) and the standard protocol (including T1-, non-fat-, and fat-suppressed T2-weighted sequences), for the assessment of spinal non-contrast MRI in the sagittal plane in patients with non-specific low back pain and/or lumbar radiculopathy. The interchangeability of the two protocols was shown using the individual equivalence index, a statistical method described to assess whether a new test can be switched with another one, particularly useful in the absence of a true reference standard, which is the case for lumbar spine MRI [27].

Second, we showed that the interreader agreement when both readers used the standard protocol was similar to the interreader agreement when one reader used the simplified protocol. Of note, the interreader agreement was variable depending on the item, from fair to substantial, but intrareader agreement was more consistently high, from substantial to almost perfect. These results are consistent with previous reports showing moderate interreader agreement for the analysis of lumbar spine MRI. In a previous study on 111 MRI examinations, with four expert readers with at least 12 years of experience, who had received training prior to the study, as well as an illustrated handbook to use during the assessment, the interreader agreement was found to be fair in assessing Modic changes and facet arthropathies [32]. The fact that the readers in our study were from two different institutions and had not received any training prior to the readings might explain the slightly lower interreader agreement in our study (moderate vs. fair). It is to note that in both studies, the intrareader agreement was substantial for these items. The moderate interreader agreement illustrates the inherent subjectivity to grade some of these items and makes difficult the use of a consensus reading as a gold standard. This, in addition to the unavailability of a surgical gold standard, formed the rationale for testing the interchangeability of the protocols, as discussed above.

Third, we showed that the rate of findings was not statistically different between the protocols, except for focal bone marrow abnormalities, foraminal stenosis, and facet arthropathy, for which the rate of findings was higher with the simplified protocol. The higher rate of findings on the simplified protocol for focal bone marrow abnormalities is consistent with that of prior studies showing a higher contrast-to-noise ratio of fat-only images compared with that of T1-weighted images for the detection of bone marrow metastases and focal bone marrow lesions in multiple myeloma [13, 33]. Fat deposits around sacroiliac joints in the context of spondylarthritis were also shown with higher contrast-to-noise ratio on fat-only images compared with that on T1-weighted sequences [11]. The better delineation of foraminal fat (and its disappearance in case of foraminal stenosis) on fat-only images compared with that on T1-weighted images probably explains the higher rate of findings on the simplified protocol. This higher rate of findings on the simplified protocol may however reflect a higher rate of false-positives (rather than higher sensitivity) with the fat-only images. Future studies should investigate the correlation between foraminal stenosis as detected on fat-only images and neurological manifestations. Finally, it is not clear why facet arthropathy was more often reported on the simplified protocol. Active facet arthropathy showing inflammatory changes, arguably the most relevant clinically, is detected on fat-suppressed T2-weighted images that were common to both protocols [34]. Chronic facet arthropathy may present periarticular fatty deposits that could be more readily detected on fat-only images due to higher contrast-to-noise ratios. However, we did not distinguish between these forms of facet arthropathy in this study and the performance of Dixon sequences in this indication should be investigated in future studies.

Overall, our results suggest that the acquisition of T1-weighted sequences could be waived in the workup of non-specific low back pain and lumbar radiculopathy, providing an opportunity to decrease the acquisition time. In fact, the average acquisition time for the standard protocol (T1- and T2-weighted sequences) was 6 min 49s ± 0 min 65 s, compared with 3 min 59 s ± 0 min 41 s for the simplified protocol. It is to note that a T2-weighted Dixon sequence may be used to replace the whole set of the three sequences recommended in the sagittal plane in lumbar spine MRI (a T1-, a T2-weighted, and a fat-suppressed fluid-sensitive sequence). Prior to this study and based on previous literature, we had already included the Dixon sequence in our spine protocol and were using the combination of T2-weighted and fat-suppressed T2-weighted images that the Dixon sequence provides [10, 18]. In practice, the use of a T2-weighted Dixon sequence in place of the three standard sequences therefore would allow a more significant decrease of acquisition time than what is reported in this study. A shorter acquisition time in the sagittal plane may benefit to the patient’s comfort. Additional sequences that may provide useful information on the cause of the patient’s symptoms can also be obtained in the time saved, including a coronal fat-suppressed fluid-sensitive sequence on the lumbar spine and pelvis, as advocated by some authors [35].

In practice, T1-weighted images have since long been included in MRI protocols of the spine to assess a variety of findings, which can be divided into morphological and signal abnormalities. T1-weighted sequences may be typically used for the morphological assessment of spinal structures. While fat-only images lack the anatomical conspicuity of T1 lesions, the non-fat-suppressed in-phase images and the fat-suppressed water-only images provided by the T2-weighted Dixon sequence do allow the assessment of morphological abnormalities. This was confirmed by our results through the interchangeability of the simplified and standard protocols for the detection of degenerative changes at the vertebral margins, Schmorl’s nodes, and vertebral fractures. Additionally, T1-weighted sequences are more specifically used for the assessment of the signal of fat. They serve to detect and characterize areas of increased or decreased bone marrow fat content. Fat, a major component of bone marrow, is hyperintense on T1-weighted sequences [5, 23]. Increased bone marrow fat content, or red marrow depletion (such as in Modic type 2 changes, fatty hemangiomas, or fat islands), has increased signal intensity on T1, while bone marrow replacement lesions present decreased signal compared with that of muscles or lumbar discs [5, 23]. The assessment of areas of increased or decreased fat content can be performed in the same fashion on fat-only images as on FSE T1-weighted images. However, unlike T1-weighted sequences, fat-only images are specific to the signal of fat, and the latter are therefore not subject to potential false-negatives of bone marrow replacement lesions due to other short T1 substances (including melanin, blood, proteinaceous fluids, and paramagnetic substances). The interpretation of fat-only images is therefore straightforward: any areas presenting signal specifically correspond to areas containing fat, while areas where the bone marrow is void of signal correspond to areas of bone marrow replacement, as is the case for metastases [13]. This specificity to the signal of fat confers higher contrast-to-noise ratios to fat-only images in comparison with T1-weighted sequences, as discussed above [11, 13, 33].

Although fat-only images are perfectly suitable for the detection and characterization of bone marrow and fat-containing lesions, it should be kept in mind that in some circumstances, additional sequences might be needed to complement the simplified protocol, as is currently the case when using the standard protocol. In the workup of focal bone marrow replacement lesions for example, pre- and post-contrast T1-weighted sequences should be acquired. In this context, the analysis of pre-contrast T1-weighted sequences may be useful to characterize focal lesions that might contain other short T1 components such as melanin in case of metastases of melanoma. Contrast-enhanced T1-weighted sequences are also required for the assessment of spinal infectious disease. However, these indications are beyond the scope of this study, which focused on non-contrast MRI protocols in the setting of non-specific low back pain/lumbar radiculopathy.

Some limitations of our study must be acknowledged. First, our assessment was limited to sequences from a single manufacturer in one center, and although examinations were performed on three different types of scanners, they were all acquired on 3-T magnets. In our experience, FSE T2 Dixon sequences perform equally at 1.5 T, but this has to be assessed in future studies. Second, there were a relatively low number of positive cases for some items, in particular for endplate fractures and spondylolysis, accounting for the relatively large confidence intervals of kappa values. While these results should be confirmed in a larger-scale study, it is to note that fractures and spondylolysis, when symptomatic in the acute/subacute setting, commonly present high signal intensity on fat-suppressed fluid-sensitive sequences, and it is unlikely that these could be missed on a simplified protocol. Third, readers could not be blinded to the protocol being assessed, but care was taken to minimize verification and recall bias by performing the readings of the examination in a random order and by ensuring that the two protocols were read in different sessions.

In conclusion, we showed that for the workup of non-specific low back pain/lumbar radiculopathy, a simplified protocol corresponding to a single T2-weighted Dixon sequence could replace the standard protocol of lumbar spine MRI in the sagittal plane, which combines T1-weighted, non-fat-suppressed, and fat-suppressed T2-weighted sequences. Lumbar spine MRI in the sagittal plane could consequently be limited to a single sequence in this indication, providing an opportunity to reduce examination time and improve patients’ comfort.

## Electronic supplementary material

ESM 1 (DOCX 2143 kb)

## Abbreviations

CHESS: Chemical shift selective
FSE: Fast spin-echo
iPAT: Integrated parallel acquisition technique
MRI: Magnetic resonance imaging
STIR: Short-tau inversion recovery
