# Supplementary material for: MRI of non-specific low back pain and/or lumbar radiculopathy: do we need T1 when using a sagittal T2-weighted Dixon sequence?
Source: Eur Radiol. 2020 Feb 4;30(5):2583–93. doi: 10.1007/s00330-019-06626-6 (PMC7160219; doi:10.1007/s00330-019-06626-6)
Supplement: Supplementary file 1 — (DOCX 2143 kb) [file 330_2019_6626_MOESM1_ESM.docx]

**
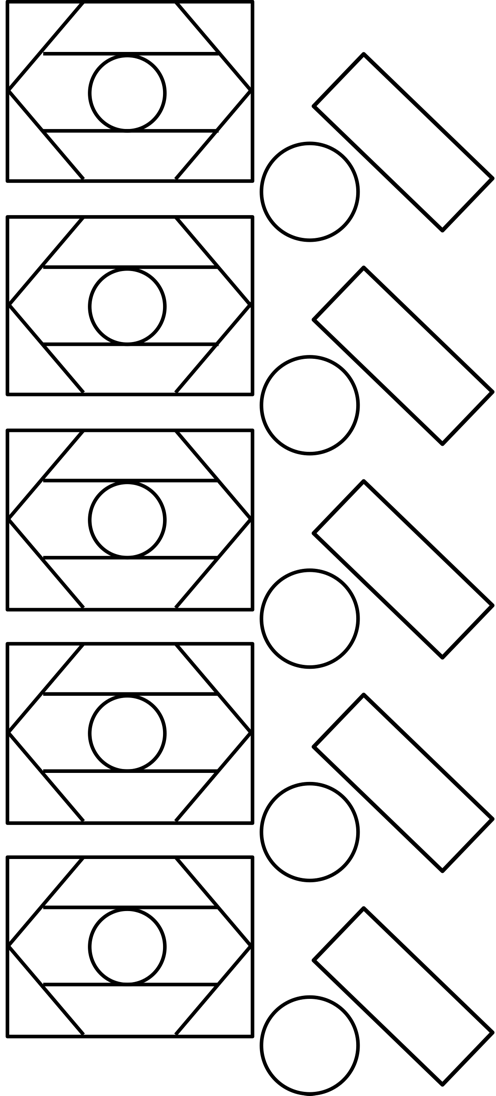
**

**Supplementary figure legend:**

Diagram used by each reader to report the findings for each lumbar vertebra (n=5), vertebral endplate (n=10), vertebral corner (n=20), facet joint (n=10), intervertebral foramen (n=10), lamina (n=10) for each examination and each protocol separately. The following items were evaluated: focal bone marrow abnormalities; juxtadiscal Modic changes, classified as inflammatory, fatty, or fibrous; degenerative changes at the margin for the vertebral bodies (including fatty changes, erosions, osteophytes); Schmorl’s nodes; facet arthropathy; spondylolysis and vertebral fractures which were reported as present or absent; and foraminal stenosis, graded as absent, mild, moderate or severe, according to a previously published grading system.
